# Highlight report: *Diploptera functata* (cockroach) milk as next superfood

**DOI:** 10.17179/excli2018-1437

**Published:** 2018-07-25

**Authors:** Kamal Niaz, Elizabeta Zaplatic, Jonathan Spoor

**Affiliations:** 1 Faculty of Bioscience and Agro-Food and Environmental Technology, University of Teramo, 64100, Italy; 2 Erasmus University Medical Centre, Erasmus University Rotterdam, Rotterdam, the Netherlands

## ⁯

Milk is a rich source of nutrients and considered by many as a valuable element of a complete diet. Traditional mammalian milk such as cow's milk contains many bioactive components that boost the physiological processes in the body. The majority of the bioactive constituents of milk are proteins such as immunoglobulins, lactoferrin and other peptides obtained from the hydrolysis of proteins. Milk however also contains fatty acids such as linoleic acid and oleic acid, minerals, different oligosaccharides and melatonin (Andreas et al., 2015[1]; Wendorff and Haenlein, 2017[15]). Apart from its well-known health benefits, milk is sometimes associated with less favourable effects. Allergy, commonly occurring in children, is one of these. Luckily this problem can be eliminated by removal of beta-lactoglobulin (β-LG). This is achieved with the help of lactic acid bacteria that are capable of hydrolysing immunoreactive proteins in milk (Biscola et al., 2018[3]). Further drawbacks to milk as a consumption good are mostly related to procedures necessary to its preservation. Over the last decades, genetic engineering has been employed in the production of novel super foods that are gradually beginning to claim their place among traditional foods. A recent study has shown that milk gained from the cockroach species *Diploptera functata* (*D. functata*) has a higher nutritional potential than conventional mammalian milk. It contains rich stores of essential nutrients such as oleic acid, conjugated linoleic acid, omega-3 fatty acids, short-chain and medium-chain fatty acids, vitamins and minerals (Banerjee et al., 2016[2]). In this editorial, light is shed on cockroach milk as a potential new super food and important alternative to traditional mammalian milk that might become available for consumption in the near future.

**Figure 1:**
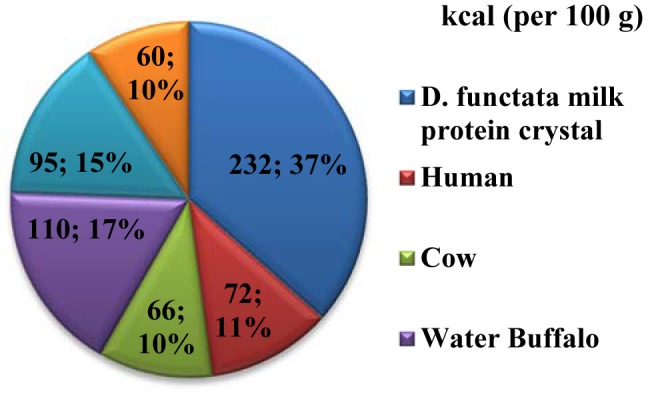
Pie chart illustrating energy values per 100 g of milk protein (kcal)

*D. functata* is a cockroach species known to give birth to live offspring. It has the ability to produce milk in the form of a substance containing protein crystals that serves as nutrition for its young. This so-called 'cockroach milk' is a source of N-acetyl-D-glucosamine, β-D-mannose, oleic acid, linoleic acid and glycerol (Banerjee et al., 2016[2]). Mass spectrometry has identified in these protein crystals the presence of four N-linked glycosylation sites, namely Ans35, Ans66, Ans79 and Ans145. This makes it structurally and functionally similar to eukaryotic proteins (Banerjee et al., 2016[2]). The main glycan is composed of two N-acetyl-glucosamine molecules and one mannose molecule. The protein crystals produced by this particular insect species (lipocalin-like milk proteins) contain energy stores equivalent to approximately 3.7 × 10^-5 ^J. This is three times (232 kcal per 100 g or 37 %) the energy content of milk produced by buffalo's and other mammals as shown in Figure 1(Fig. 1). It has been demonstrated that these crystal proteins contain large amounts of vital constituents such as fats, sugar and proteins, and are particularly rich in essential amino acids. Furthermore, it has been shown that during digestion of these crystal proteins, amino acids are released at a continuous rate (Banerjee et al., 2016[2]). Amino corrosive grouping, glycosylation and binding of unsaturated fatty acids in *D. functata* milk protein crystals are highly heterogeneous (Banerjee et al., 2016[2]). These results in a tight lipocalin overlap and shape the crystals with a firmly stuffed crystalline cross section. This previously unknown form of storage has capabilities to hold molecules more efficiently for a constant supply of nutrients essential to growth and development (Banerjee et al., 2016[2]). The high protein heterogeneity inside a single *in vivo*-developed protein crystal, accounts for the super food properties ascribed to cockroach milk. 

A recent study conducted in Brazil revealed that flour made of another breed of cockroaches, *Nauphoeta cinerea* (*N. cinerea*), has a protein content far superior to commercial flour acquired from wheat (63.22 % versus 9.8 %). This 'insect flour' contains eight essential amino acids and a high quantity of omega-3 (ω-3) and omega-9 (ω-9) fatty acids. Amino acid analysis revealed that flour produced from the *N. cinerea* cockroach contains leucine, lysine, and valine at percentages of 3.51, 3.37 and 2.61 % respectively. As for fatty acids, the flour consists of 5.69 % palmatic acid, 9.17 % oleic acid, 7.51 % saturated- and 10.94 % unsaturated fatty acids, all of them acknowledged components of a healthy diet (De Oliveira et al., 2017[5]). It has been suggested that normal wheat flower could be amplified with a supplement of cinereous cockroach flour of up to 5 %. This way *N. cinerea* flour's benefits could be effectuated without any loss of gustatory quality. Superfoods like the aforementioned cockroach milk and cockroach flour are expected to play a pivotal role in the solution to food shortage in the decades to come (De Oliveira et al., 2017[5]). 

Milk protein crystals and other products produced from cockroaches could be part of a next generation of super foods. Milk is a valued component of the human diet as its nutritional properties enhance human health and quality of life. It is known that milk contributes to the prevention of certain diseases and reduces certain threats to health. Several authors mention milk in association with a reduction in risk of cancer, ischaemic heart disease and diabetes (Elfahri et al., 2016[6]; Hayashi et al., 2015[7]; Hove et al., 2015[8]; Lamb et al., 2015[9]; Munblit et al., 2017[10]; O'Shea et al., 2000[11]; Pereira and Vicente, 2018[12]; Shori, 2015[13]; Tsuda et al., 2000[14]; Xiao et al., 2018[16]; Yang et al., 2017[17]; Chen et al., 2014[4]). Needless to say, it is unknown the extent to which cockroach milk shares these qualities with mammalian milk and that will be an important topic for future research. Further investigation should be done to reveal the exact composition of cockroach milk and the health implications of its consumption. Meanwhile, considerable effort is being put into the study of gene sequences of milk protein crystals and different biotechnological approaches are being employed for their production. The importance of the identification of protein sequences like that of cockroach milk, can hardly be overestimated. Research into new nutritional strategies will contribute significantly to overcoming food shortages that are expected to arise during the lifetime of generations to come.

## Conflict of interest

There is no conflict of interest.

## Acknowledgements

The authors who worked on this manuscript acknowledge their respective universities and institutes.
